# Non-Targeted Nuclear Magnetic Resonance Analysis for Food Authenticity: A Comparative Study on Tomato Samples

**DOI:** 10.3390/molecules29184441

**Published:** 2024-09-19

**Authors:** Biagia Musio, Rosa Ragone, Stefano Todisco, Antonino Rizzuti, Egidio Iorio, Mattea Chirico, Maria Elena Pisanu, Nadia Meloni, Piero Mastrorilli, Vito Gallo

**Affiliations:** 1 Department of Civil, Environmental, Land, Building Engineering and Chemistry (DICATECh), Polytechnic University of Bari, Via Orabona, 4, I-70125 Bari, Italy; rosa.ragone@poliba.it (R.R.); stefano.todisco@poliba.it (S.T.); antonino.rizzuti@poliba.it (A.R.); piero.mastrorilli@poliba.it (P.M.); vito.gallo@poliba.it (V.G.); 2 Istituto Superiore di Sanità, Core Facilities Istituto Superiore Di Sanità, Viale Regina Elena, 299, I-00161 Roma, Italy; egidio.iorio@iss.it (E.I.); mattea.chirico@iss.it (M.C.); mariaelena.pisanu@iss.it (M.E.P.); 3 Agenzia Regionale Protezione Ambientale Lazio, Dipartimento Prevenzione e Laboratorio Integrato, Servizio Coordinamento delle Attività di Laboratorio, Unità Laboratorio Chimico di Latina, Via Mario Siciliano, 1, I-04100 Latina, Italy; nadia.meloni@arpalazio.it; 4 Innovative Solutions S.r.l., Spin-Off Company of the Polytechnic University of Bari, Zona H 150/B, I-70015 Noci, Italy

**Keywords:** metabolomic analysis, NMR, geographical origin, method validation, fingerprint, inter-laboratory comparison, food control, traceability

## Abstract

Non-targeted NMR is widely accepted as a powerful and robust analytical tool for food control. Nevertheless, standardized procedures based on validated methods are still needed when a non-targeted approach is adopted. Interlaboratory comparisons carried out in recent years have demonstrated the statistical equivalence of spectra generated by different instruments when the sample was prepared by the same operator. The present study focused on assessing the reproducibility of NMR spectra of the same matrix when different operators performed individually both the sample preparation and the measurements using their spectrometer. For this purpose, two independent laboratories prepared 63 tomato samples according to a previously optimized procedure and recorded the corresponding 1D ^1^H NMR spectra. A classification model was built using the spectroscopic fingerprint data delivered by the two laboratories to assess the geographical origin of the tomato samples. The performance of the optimized statistical model was satisfactory, with a 97.62% correct sample classification rate. The results of this work support the suitability of NMR techniques in food control routines even when samples are prepared by different operators by using their equipment in independent laboratories.

## 1. Introduction

In the last decades, applications of metabolomics based on NMR spectroscopy for food control, in terms of quality, safety, and authenticity, have extraordinarily increased [1,2,3,4,5,6,7,8,9], and many attempts have been made to include NMR protocols in the list of the official routine methods [10,11,12]. Due to the chemical complexity that generally characterizes food matrices, the non-targeted approach (combined with multivariate statistical analysis) finds many applications in studies on the variety, geographical origin, possible frauds, production, and industrial processing of foodstuffs [13,14,15,16,17]. The non-targeted approach allows getting a large amount of information on the wide range of metabolites (fingerprinting) or a set of selected metabolites (profiling, often referred to as the semi-targeted approach) contained in a complex mixture [16,18,19,20,21].

In this context, metabolomic analysis based on ^1^H NMR spectroscopy presents multiple advantages: it is non-destructive, intrinsically quantitative, not time-consuming, cost-effective, and particularly robust [22].

Specifically, the robustness of ^1^H NMR spectroscopy is based on the ability to generate comparable spectra for the same set of samples using differently configured instruments. The results of some recent interlaboratory studies demonstrated that the statistical equivalence of NMR spectra can be reached when the FIDs, generated by differently configured spectrometers upon applying optimized acquisition parameters, are opportunely processed. Indeed, inter-laboratory variance can be significantly decreased when the spectra are processed by a single operator compared to multi-operator processing regardless of the software used [23,24]. Furthermore, it has been demonstrated the crucial importance of the method adopted to reduce the raw spectral data to the numerical matrix subsequently employed for the development of a classification model [25,26,27,28].

However, these studies did not account for the variability strictly related to sample preparation. To the best of our knowledge, few works report on comparative studies in which each participating laboratory executed the entire analytical protocol from sample management to NMR acquisition. Deborde et al. focused on the standardization of plant extract preparation, setup of NMR instruments (400, 500, and 600 MHz), verification of sample spectra quality, and spectra processing steps. Specifically, the quality of the produced spectra was based on the coefficient of variation in the full width at half maximum (FWHM) and the signal-to-noise ratio (S/N) of two selected peaks (finding values comprised between 5 and 10% depending on the size of the sample set and the spectrometer field). The global variance of the spectra was verified through the score distance in the PCA scores plot along the components PC1 and PC2 [29].

Herein, further steps have been made to demonstrate the robustness and the applicability of NMR data to generate comparable spectra even when the samples are prepared and analyzed independently by two different laboratories using their equipment and following established protocols. First, the protocols for the sample preparation and NMR measurement were optimized based on statistical quality parameters. Next, the statistical equivalence of the pool of spectra produced by the two laboratories was assessed by applying a multivariate data analysis. Ultimately, a classification model was built and validated to discriminate between tomato samples cultivated in two different Italian regions, namely Lazio and Sicily. Details on the optimization of the sample preparation protocol, along with statistical considerations to assess the comparability of the generated spectra and the reliability of the constructed classification model, will be discussed throughout the manuscript.

## 2. Results and Discussion

### 2.1. Optimization of Sample Preparation Protocol

Three different protocols for the sample preparation were tested to evaluate both the extraction capability and the sample stability over the analysis time.

A detailed description of the three protocols is reported in Section 4.1.1, Section 4.1.2 and Section 4.1.3, starting from mechanically squeezed tomatoes (P1) [30], followed by lyophilized tomatoes (P2) [31,32,33,34], and homogenized tomatoes (P3) [35]. The choice of the most suitable sample preparation protocol was based on two experimental evaluations, namely the extraction capability and the repeatability of the NMR measurements. The extraction capability was verified through the analysis of the metabolic composition of the aqueous extracts obtained upon application of the three different protocols. Spectroscopic investigations suggested that a higher number of metabolites, such as amino acids and carbohydrates, could be extracted from P3 (See Figure S1 in Supplementary Material). Additionally, the precision and repeatability of the three analytical protocols, including sample preparation and 1D ^1^H NOESY (Nuclear Overhauser Effect Spectroscopy) measurement, were evaluated. For this purpose, ten NMR samples were prepared for each protocol using the same batch of tomatoes. A well-defined signal in the 1D ^1^H NOESY spectrum at 1.32 ppm was selected, and the area under such a peak was calculated in the ten spectra. The signal selection was based on its satisfactory resolution and the observed stability of the corresponding metabolite over time. In the absence of changes in the NMR spectra, as in the present case, the calculation of the relative standard deviation of a single signal has been demonstrated as a valid parameter to evaluate the repeatability of NMR measurements [23]. The obtained integral values were subjected to descriptive statistics. As shown in Table 1, the lowest value of %RSD was found when P3 was applied for the sample preparation (%RSD = 1.32), suggesting that integral values calculated for the ten prepared samples were more tightly clustered around the mean compared to the cases P1 and P2. Based on the extracting capability and the measurement reliability, P3 was selected as the most suitable sample preparation protocol for this study and, therefore, was followed by the two laboratories for the subsequent NMR measurements.

### 2.2. Metabolic Profile of Tomatoes Aqueous Extracts Following P3

**Figure 1:**
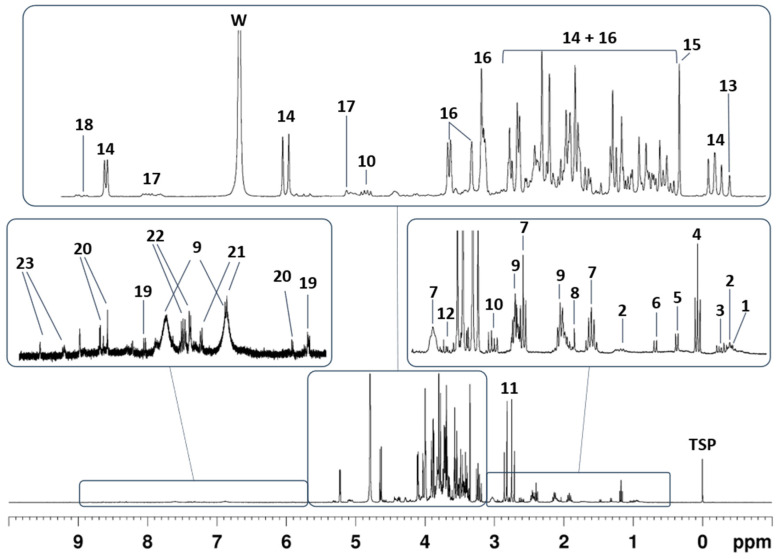
A typical 1D ^1^H NOESY spectrum of an aqueous extract of a tomato sample (400 MHz). The main classes of metabolites identified via comparison with reference compounds are indicated by increasing numbering. The full chemical shift assignment is reported in Table 1. “W” refers to the residual water signal. The chemical shift scale is referenced to the TSP-*d*_4_ singlet at 0 ppm.

The main classes of water-soluble metabolites were identified by comparing the 1D ^1^H NOESY spectra of the aqueous extracts from homogenized tomatoes (Figure 1) with those of reference compounds.

The main classes of metabolites detected in the 1D ^1^H NOESY spectra of the samples under investigation are in agreement with the data reported in the literature [36,37]. They include organic acids as acetic, malic, and citric acids (**7**, **10**, and **11** in Figure 1); carbohydrates as glucose, fructose, and galacturonate (**14**, **16**, **17**, and **18** in Figure 1); amino acids as isoleucine, leucine, valine, threonine, alanine, GABA, glutamine, aspartic acid, tyrosine, and phenylalanine (**1**, **2**, **3**, **5**, **6**, **8**, **9**, **12**, **21**, and **22** in Figure 1); alcohols as ethanol and methanol (**4** and **15** in Figure 1); nucleosides as adenosine and uridine (**19** and **20** in Figure 1); quaternary ammonium compounds as choline and trigonelline (**13** and **23** in Figure 1). The complete list of metabolites contained in the aqueous extracts of tomato as identified via 1D ^1^H NOESY measurements is reported in Table S1 in Supplementary Material. Characterization was based on literature data and comparison with reference compounds. Among the attributed signals, those at 6.88 and 7.60 ppm were addressed (Figure 1, compound **9**). Based on the comparison with the reference solution, such signals were assigned to the hydrogen atoms of the amide group of glutamine (**9**). Interestingly, such signals are not observed in the NMR spectra reported in the public database when glutamine is dissolved in water or deuterated water at pH = 7 [38]. Conversely, under our conditions (pH = 4.2), the two broad resonances were observed both in the tomato extracts under investigation and a reference solution of pure glutamine. ^1^H-^15^N HMQC experiment performed on a reference solution of pure glutamine revealed that the two signals belong to hydrogen atoms bound to the same nitrogen atom at 112 ppm (see Figure S2 in Supplementary Material). Based on the ^15^N chemical shift value reported in the literature, such nitrogen can be attributed to the amide functional group [39]. In addition, an exchange process between these two hydrogen atoms was observed performing a 2D ^1^H NOESY-EXSY experiment (see Figure S3 in Supplementary Material). The exchange with water, if any, could not be detected due to suppression of the water signal during the acquisition of the NMR measurement. Moreover, a NOE contact was observed between the signal at 7.60 ppm and the signal at 2.45 ppm (-CγH2-), suggesting a *syn* relation between these hydrogen atoms. Based on these findings, it can be argued that the rate of the rotation around the C-N in the amide moiety is slowed down under our conditions (pH = 4.2), likely due to a stabilization of the iminol form in the tautomeric equilibrium of the amide. The same considerations were made for the aqueous extracts of tomatoes based on the information contained in the 2D ^1^H NOESY-EXSY experiment (see Figure S4 in Supplementary Material).

### 2.3. Statistical Analysis

**Figure 2:**
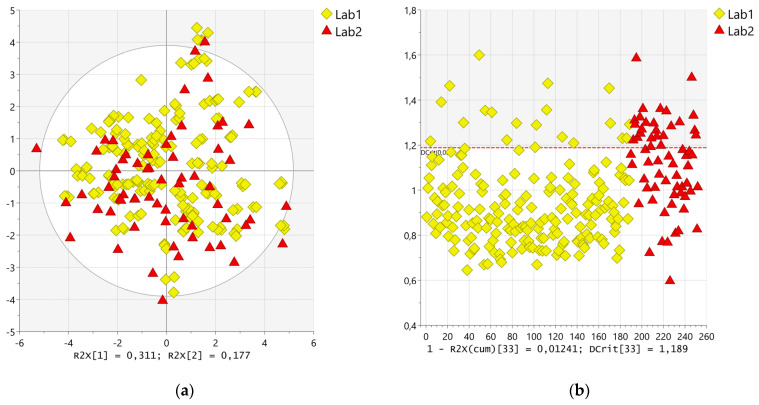
(**a**) PC1/PC2 scores plot related to PCA and (**b**) DModX plots using a dataset composed of all the spectra registered by Lab1 and Lab2. The observations are indicated as yellow diamonds and red triangles for spectra produced by Lab1 and Lab2, respectively.

The numerical matrix obtained from the data treatment described in Section 4.3 was imported into SIMCA 17.0.2 software. A statistical investigation was carried out to assess the comparability of the spectra produced by the two laboratories (Lab1 and Lab2) when they independently prepared the tomato samples according to a well-defined protocol through their equipment. A PCA was performed on the spectral data after a Pareto scaling, which resulted in the most suitable one, to evaluate the sample distribution of the whole dataset containing the spectra recorded by both Lab1 and Lab2. As illustrated in the scores plot (Figure 2a), no significant distance was observed between the group of spectra produced by Lab1 (yellow diamond) and the group of spectra delivered by Lab2 (red triangle). To confirm such a hypothesis, once PCA was applied, the DModX scores plot (Figure 2b) was investigated, assessing that all the spectra produced by the two laboratories were located within the 2-fold DCrit value (2 × 1.189).

To further confirm the similarity among the spectra produced for the same samples by the two laboratories, a cluster analysis was performed by adopting Euclidean as the Distance Measure and Ward as the Clustering Algorithm. As illustrated in the hierarchical clustering dendrogram (see Figure S5 in Supplementary Material), generally, the spectra produced by the two laboratories for the same sample clustered very closely. Moreover, a pair-wise correlation was attempted, selecting samples as a dimension, Kendall rank correlation as a distance measure, and a correlation cutoff equal to 0.05. The values of correlation coefficients were generally higher than 0.50 both for the replicates produced by Lab1 for the same sample and for the spectra produced by both the laboratories for the same sample (see Figure S6 and Table S2 in Supplementary Material for further details). The evidence obtained from these statistical investigations suggested a satisfactory comparability of the recorded spectra both intra-laboratory (short distance among the replicates produced by Lab1) and inter-laboratory (short distance among the spectra produced by the two laboratories for the same sample).

Once the comparability among the 1D ^1^H NOESY spectra produced by the two laboratories was demonstrated, the resulting dataset was exploited to build an optimized classification model to assess the geographical origin of the tomato samples. For this purpose, OPLS-DA was adopted as a supervised statistical approach to discriminate between two groups of samples cultivated and collected from Sicily (26 samples) and Lazio (37 samples), respectively. As summarized in Table 2, different combinations of training and validation sets were adopted to verify possible effects on the reliability and prediction capability of the resulting classification models (M1–M9). In all cases, the ratio of samples according to the belonging class, namely Sicily (SI) and Lazio (LA), was kept uniform within the training and the validation sets (ratio SI/LA ≈ 1.4).

In the first three cases, two sets of spectra produced by the two laboratories were used to train and validate the models. In the very first case (M1), the training set was composed of 126 out of 189 spectra produced by Lab1 (considering all the replicates) and 42 out of 63 spectra produced by Lab2, randomly extracted from the available spectra (ratio 3:1). The same ratio of 3:1 was maintained for the validation, being formed of 63 spectra from Lab1 and 21 ones from Lab2. Next, to evaluate the effect of the replicates on the overall performance of the classification model, the three replicates produced by Lab1 for each sample were merged based both on the average and median values to train and validate M2 and M3, respectively. Specifically, the merged spectra were used as a training set and validation set in a ratio of 2:1 in M2 as average spectra (Lab1 avg) and in M3 as median spectra (Lab1 med). Ultimately, to verify the interchangeability of the spectra regardless of the producing operator/equipment, four further models, M4–M9, were built and evaluated. In the case of M4, all the spectra produced by Lab1 (189) were used as a training set, while all the spectra recorded by Lab2 (63) were employed to test the model. The average spectrum and the median one were used to train M5 and M6, respectively. In these cases, the spectra produced by Lab2 were employed to validate the models. Lastly, a model was trained by using the 63 spectra recorded by Lab2. Such a model was validated against three different sets composed of the spectra produced by Lab1. Specifically, all spectra, including the replicates, were used in M7; the average spectra were employed in M8 and the median spectra in M9.

Table 3 summarizes the main quality parameters of the nine classification models (M1–M9). The number of predictive (P) and orthogonal components (O) and the total explained variance both in the X and Y matrices were evaluated to assess the main features of the developed model.

M1 and M4 resulted in the most stable and reliable models, being characterized by the highest values of R^2^Y (cum) and Q^2^ (cum) values, i.e., R^2^Y (cum) = 0.767 and Q^2^ (cum) = 0.619 for M1; R^2^Y (cum) = 0.802 and Q^2^ (cum) = 0.718 for M4. The models M2 and M3 showed equally satisfactory values of R^2^Y (cum) and Q^2^ (cum) values, being around 0.741–0.748 and 0.505–0.521, respectively. Conversely, the quality of the remaining models (M5–M9) was not satisfactory, with R^2^Y (cum) and Q^2^ (cum) values of 0.505–0.567 and 0.308–0.419, respectively.

The statistical significance of the models was evaluated by computing the *p* values upon application of analysis of variance (CV-ANOVA) in the cross-validated residuals of the Y-variables. The lowest *p*-values were obtained for M1 (*p* = 4.9424 × 10^−23^) and M4 (*p* = 1.4347 × 10^−37^), confirming the highest reliability of these two models.

Permutation tests were performed to check the degree of overfit for the OPLS-DA (number of permutations = 200), whereas the Q^2^ intercept value < 0.05 and R^2^ intercept value < 0.40 were assumed as indicative of a valid model. As described in Table 3, the computed Q^2^ and R^2^ intercepts suggested that all the built models can be considered valid and correctly fitted (see Figure S7 in Supplementary Material for further details).

Once the models were fitted on the training sets and the reliability was assessed, the models were used to predict the responses for the observations in the validation set (see Figure S8 in Supplementary Material for further details). The models M1–M3 fitted on a mixture of samples produced by the two laboratories were able to achieve a high correct prediction of the observations in the validation set (90.48–97.62%). In all the other cases (M4–M9), the prediction capability was lower, with a percentage of correct predictions in the range of 80.95–87.30%.

**Figure 3:**
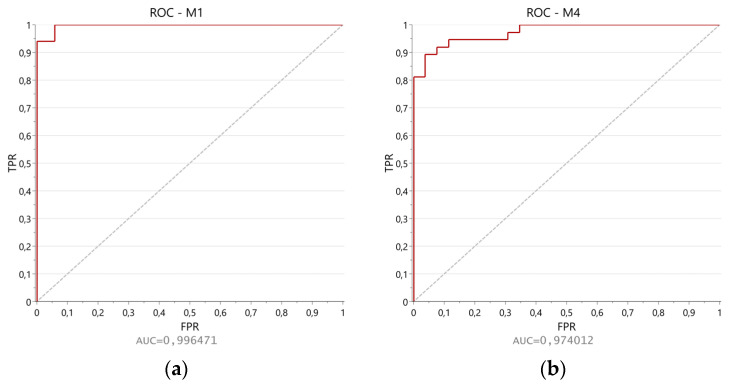
ROC curves for models (**a**) M1 and (**b**) M4.

The Receiver Operating Characteristic (ROC) curves were investigated to evaluate the difference in the performance of M1 and M4, which have resulted in the most promising models according to the quality parameters illustrated in Table 3 and the results of CV-ANOVA. The values of Area Under the Curve (AUC) were computed both for M1 (Figure 3a) and M4 (Figure 3b), finding a higher value for the first model (0.996471) compared to the latter (0.974012).

## 3. Discussion

The results of the present study represent a step forward and an integration of the previous ones reporting on the aspects affecting the proficiency of inter-laboratory comparisons dealing with the analysis of food matrices through NMR-based metabolomics. The evaluation of the statistical equivalence of NMR spectra produced by different facilities has been the object of many inter-laboratory comparisons, both for the quantification of well-defined metabolites [40,41,42,43] and for screening purposes towards the assessment of food authenticity and traceability [23,44,45]. The present work aimed at establishing the comparability of the 1D ^1^H NOESY spectra produced by two different laboratories during the analysis of food samples (tomatoes) and the applicability of such spectroscopic data for the construction of an opportune classification model able to discriminate, as a case study, between tomato samples cultivated in two Italian regions, i.e., Lazio and Sicily.

The preparation protocol was deeply investigated in a way to achieve a good compromise in terms of extraction capability, which translates into the amount of metabolites extracted from tomatoes, and satisfactory measurement repeatability. For this purpose, three different preparation protocols were investigated. Following an in-depth analysis of the 1D ^1^H NOESY spectra, the aqueous extract of homogenized tomato was found to be the most informative in terms of the number of extracted metabolites compared to that obtained from tomato juice or lyophilized tomato. In addition, sample preparation starting from homogenized tomatoes allowed for more repeatable measurements, considering the relative standard deviation (%RSD) computed for the integral of well-defined signals in spectra of ten replicates of the same tomato batch. The optimized protocols for the sample preparation and NMR measurement were performed by two different laboratories utilizing their facilities. An in-depth statistical investigation was carried out to establish the comparability of the produced spectra for the same sample. Specifically, the distance among the spectra was studied by analyzing the distance of the samples within a PCA model (DModX), the distance among the samples according to hierarchical clustering, and the values of the correlation coefficients among the samples. Based on the results of these investigations, the spectra produced by the two laboratories showed a reciprocal very short distance, confirming a satisfactory statistical equivalence among them. The pool of spectra was then used for the construction of the OPLS-DA model to discriminate the tomato samples cultivated in Lazio from those cultivated in Sicily. Different combinations of training and validation sets were investigated, aiming at obtaining a reliable and valid classification model. The best results were observed when a combination of spectra produced by the two laboratories was used to train the model (M1). In this case, the OPLS-DA model resulted as highly reliable and fitted in terms of quality parameters and validation tests. Importantly, this model was found to be highly predictive with a correct prediction rate of around 97% when a mixture of 84 samples produced by both laboratories and unknown to the algorithm was used to query the model (Table 4, M1). Lower performance was observed when the model was trained using all the spectra produced by a single laboratory, and the model was validated using the spectra delivered by the second laboratory (Table 4, M4, and M7). Merging replicates in the form of average (Table 4, M2, M5, and M8) or median (Table 4, M3, M6, and M9) spectra resulted in a slight loss of information with a consequent deterioration in the predictive ability of the model.

The non-targeted approach utilized in this study offers several practical advantages for the food industry. Firstly, its ability to detect a wide range of compounds without prior knowledge allows for a more comprehensive monitoring of food products. This holistic view can enhance the detection of food fraud, ensuring that food products meet regulatory standards and consumer expectations. Secondly, the efficiency of the non-targeted approach in processing large datasets and identifying unique chemical fingerprints could be valuable for establishing traceability systems that are more robust and less dependent on predefined markers. This adaptability is particularly important in a globalized food market, where supply chains are complex and the types of fraud or contamination can vary significantly.

By integrating non-targeted approaches into standard traceability practices, the food industry can develop more resilient and adaptive systems that better respond to emerging challenges and risks, thereby safeguarding public health and maintaining market integrity.

The results described in this work are very encouraging, considering the possibility of building a model trained by a pool of spectra produced by independent laboratories around the world analyzing samples prepared according to a well-established protocol through their facilities. Such data collection may serve for other spectra whose authenticity is unknown to be queried for classification purposes.

## 4. Materials and Methods

### 4.1. Materials

3-(Trimethylsilyl)-2,2,3,3-tetradeutero-propionic acid sodium salt (TSP-*d*_4_, CAS N. 24493-21-8, 99%D, Armar Chemicals, Döttingen, Switzerland), hydrochloric acid (HCl, 37%w, CAS N. 7647-01-0; ≥99.5%, Sigma-Aldrich, Milan, Italy), sodium oxalate (Na_2_C_2_O_4_, CAS N. 62-76-0; ≥99.5%, Sigma-Aldrich, Milan, Italy), sodium azide (NaN_3_, CAS N. 26628-22-8; ≥99.5%, Sigma-Aldrich, Milan, Italy), and deuterium oxide (D_2_O, CAS. N. 7789-20-0, 99.86%D, Eurisotop, Saclay, France) were used for sample preparation. Methanol-*d*_4_ (CD_3_OD, CAS. N. 811-98-3, 99.80%D, Eurisotop, Saclay, France) was used for temperature calibration. Whatman syringe membrane filters (PTFE, pore size 0.2 µm, diam. 47 mm) were provided by Sigma-Aldrich. NMR tubes (Wilmad WG-1000-7) were provided by ATS Life Sciences Wilmad, Vineland, NJ, United States. A total number of 63 tomato samples were provided by ARPA-Lazio, Rome, Italy. The samples were collected from two different Italian geographical regions (n. 26 samples from Sicily and n. 37 samples from Lazio). All samples were collected according to official recommendations for sampling (Regulations (CE) no. 834/2007, no. 889/2008, no. 1235/2008, and following modifications) [46,47] and processed (homogenized) according to Regulations (CE) no. 401/2006, no. 178/2010, and following modifications [48,49].

The preparation protocol was optimized on tomatoes cv Centus F1—ISI 25765 provided by “Azienda Agricola Tulipa S.r.l.”, Stornarella (FG, Italy). Tomatoes were stored at –20 °C until analysis. The following three procedures were tested for the sample preparation: manual squeezing (P1), lyophilization (P2), and homogeneization (P3). For all three procedures, the same buffer solution of sodium oxalate [(HC_2_O_4_)^−^/(C_2_O_4_)^2−^ 0.11 M, pH 4.2] containing NaN_3_ as a bacteriostatic agent was used for the extraction. The buffer was prepared according to the following recipe: Na_2_C_2_O_4_ (37 g) was dissolved in distilled water (800 mL) at room temperature. HCl 37%w (about 6.9 mL) was added dropwise, adjusting the pH to a value of 4.2. Next, NaN_3_ (170 mg/L) was added. Finally, water was added to the solution to reach a final volume of 1 L.

#### 4.1.1. Method P1 [30]

Tomatoes were defrosted for 60 min at room temperature. Samples were mechanically squeezed and centrifuged for 10 min at 4000× *g*. An aliquot of supernatant (318 µL) was mixed with sodium oxalate buffer (222 µL) and TSP-*d*_4_/D_2_O (60 µL). The resulting solution was transferred into an NMR tube.

#### 4.1.2. Method P2 [50]

Tomatoes were freeze-dried at −50 °C and 0.045 atm for 24 h in a lyophilizer (Martin-Christ GmbH, Model Alpha 1-4 LSC, Osterode am Harz, Germany), then they were ground manually employing a mortar and a pestle. The obtained powder was sieved (through a metallic sifter, pore size of 0.5 mm) and stored at room temperature under vacuum in a plastic bag protected from light until analysis. A quantity of 200 mg of sample was weighed and dissolved in 1.8 mL of buffer. The solution was sonicated for 10 min at 40 kHz, vortexed for 5 min at 2500 rpm, and centrifuged for 10 min at 4000× *g*. A volume of 540 μL of supernatant was transferred into an NMR tube and added to 60 μL of TSP-*d*_4_/D_2_O solution. The resulting solution was transferred into an NMR tube.

#### 4.1.3. Method P3 [29]

Tomatoes were homogenized and stored at −20 °C. Before analysis, samples were thawed for 60 min at room temperature. An aliquot of 4.0 g of sample was dissolved in 1.0 mL of buffer and vortexed for 5 min at 2500 rpm. The solution was subjected to a thermic treatment for 10 min at 80 °C in a thermostatic bath, followed by a centrifugation step for 10 min at 4000× *g*. The supernatant was filtered off using a syringe filter (PTFE, porosity = 0.2 µm, diameter 47 mm). An aliquot of the filtrate (540 µL) was mixed with TSP/D_2_O (60 µL), and the resulting solution was transferred into an NMR tube.

### 4.2. NMR Measurements

NMR experiments were conducted at 298.1 ± 0.1 K. Before NMR acquisition, the temperature was calibrated using a tube containing pure methanol-*d*_4_ (CD_3_OD, 99.80%D) [51].

Lab1 used a Bruker Avance 400 spectrometer equipped with a 5 mm inverse broad-band (BBI) probe, an autosampler, and an automatic system for tuning and shimming, using TOPSPIN 3.0 software for acquisition (Bruker BioSpin GmbH, Rheinstetten, Germany). Lab2 used an NMR spectrometer Bruker Avance 400 equipped with a 5 mm inverse broad-band (BBI) probe and a manual system for tuning and shimming, using the XWIN-NMR 3.5 operative system for acquisition.

The following acquisition parameters were optimized and adopted to record the 1D ^1^H NOESY measurements: pulse program = noesygppr1d; size of fid (TD) = 128 K data points; spectral width (SW) = 20 ppm; transmitter offset = 4.70 ppm; dummy scans (ds) = 4; number of scans (ns) = 64; acquisition time = 8.12 s; mixing time (d8) = 0.01 s; recycle delay (d1) = 30 s. Before the analysis, each laboratory performed a calibration of the 90° pulse on the sample solution.

The 1D ^1^H NOESY FIDs (252) produced by the two different laboratories (189 FIDs by Lab1 and 63 by Lab2) were processed by a single operator using the software MestReNova 11.0 (Mestrelab Research SL, Santiago de Compostela, Spain). The FIDs were zero-filled with 128 K number of points before undergoing a Fourier transformation by applying an exponential multiplication function with a line broadening of 0.1 Hz. The spectrum phase was manually corrected, while the baseline was subjected to an automatic correction. The TSP-*d*_4_ singlet signal was set at δ = 0.00 ppm and used as a chemical shift reference.

### 4.3. Data Preprocessing and Chemometrics

The raw data (FIDs) derived from the 1D ^1^H NOESY measurements were processed by a single operator using MestReNova and segmented into regular-sized (0.04 ppm) intervals (buckets) in the range of [10.50, 0.50] ppm. The underlying area of each bucket was calculated and normalized to the total intensity. The areas of the buckets in the region [5.13, 4.69] ppm, corresponding to the residual water signal, were set to 0. The data were stored as a table with one sample per row and one variable (bucket) per column. The data matrices were imported into the software SIMCA 17.0.2 (Umetrics, Umea, Sweden) or MetaboAnalyst 5.0. The NMR spectra constituted the observations, while the buckets constituted the *x*-variables. Buckets were centered and subjected to Pareto scaling (each *x_j_*-variable was scaled to 1/sqrt(sd*_j_*), where sd*_j_* is the standard deviation of the *x_j_*-variable computed around the mean) to avoid noise inflation. An unsupervised method, the Principal Component Analysis (PCA), was applied to obtain an overview of all available data. Then, a supervised approach, the Orthogonal Partial Least Squares Discriminant Analysis (OPLS-DA), was performed to build a model for the classification of tomato samples according to the geographical origin, Lazio (LA) vs. Sicily (SI). The evaluation of the quality of OPLS-DA models was based on the parameters *R*^2^ (goodness-of-fit) and *Q*^2^ (goodness-of-prediction in 7-fold cross-validation). Permutation tests were performed to assess the performance of the OPLS-DA models.

## Figures and Tables

**Table 1:** Results of the descriptive statistics applied on the integral values of the signal at 1.32 ppm as contained in ten 1D ^1^H NOESY spectra recorded for ten analytical samples of the same tomato when the three protocols (P1–P3) were applied.

Statistic Parameter	P1	P2	P3
Median	0.202	0.142	0.303
Mean (μ)	0.200	0.142	0.304
Standard deviation (σ)	0.005	0.003	0.004
%RSD ^a^	2.68	1.83	1.32

^a^ Relative Standard Deviation percentage calculated as μ/σ × 100.

**Table 2:** Summary of the composition of the training set and validation set used to build the OPLS-DA models (M1–M9).

Model	Training Set	Validation Set
M1	Lab1 = 126	Lab1 = 63
	Lab2 = 42	Lab2 = 21
	Tot. = 168	Tot. = 84
M2	Lab1 avg = 42	Lab1 avg = 21
	Lab2 = 42	Lab2 = 21
	Tot. = 84	Tot. = 42
M3	Lab1 med = 42	Lab1 med = 21
	Lab2 = 42	Lab2 = 21
	Tot. = 84	Tot. = 42
M4	Lab1 = 189	Lab2 = 63
M5	Lab1 avg = 63	Lab2 = 63
M6	Lab1 med = 63	Lab2 = 63
M7	Lab2 = 63	Lab1 = 189
M8	Lab2 = 63	Lab1 avg = 63
M9	Lab2 = 63	Lab1 med = 63

**Table 3:** Summary of quality parameters of OPLS-DA models (M1–M9).

Model	No ^a^	R^2^X (cum) ^b^	R^2^Y (cum) ^c^	Q^2^ (cum) ^d^	Q^2^ Intercept	R^2^ Intercept	F-Value ^e^	*p*-Value ^f^	Correct Prediction
M1	1P + 8O	0.850	0.767	0.619	−0.444	0.254	13.4641	4.9424 × 10^−23^	97.62%
M2	1P + 6O	0.811	0.741	0.505	−0.630	0.365	5.0329	2.3550 × 10^−6^	90.48%
M3	1P + 6O	0.810	0.748	0.521	−0.645	0.371	5.3575	9.2400 × 10^−7^	92.86%
M4	1P + 8O	0.861	0.802	0.718	−0.351	0.193	24.0347	1.4347 × 10^−37^	87.30%
M5	1P + 2O	0.608	0.567	0.419	−0.357	0.215	6.7444	2.0513 × 10^−5^	80.95%
M6	1P + 2O	0.606	0.555	0.399	−0.361	0.223	8.9320	1.4372 × 10^−13^	80.95%
M7	1P + 2O	0.565	0.505	0.308	−0.363	0.242	4.1578	1.5945 × 10^−3^	85.19%
M8									85.71%
M9									85.71%

^a^ No indicates the number of predictive (P) and orthogonal (O) components. ^b^ R^2^X (cum) represents the total (predictive and orthogonal) explained variance in model samples (X). ^c^ R^2^Y (cum) represents the cumulative explained variation in the Y matrix. ^d^ Q^2^ (cum) describes the predictive ability of the model based on sevenfold cross-validation. ^e^ F-value was obtained from the F-test based on the ratio MS Regression/MS Residual (where MS stays for Mean Squares). ^f^ *p*-value indicates the probability level where a model with this F-value may be the result of just chance. For a significant model, the *p*-value should be lower than 0.05.

**Table 4:** The Misclassification table shows the proportion of correctly classified observations in the prediction set for the OPLS-DA models (M1–M9). Correctly classified observations are indicated in green. Misclassified observations are indicated in pink.

Model	Class	Prediction Set	Predicted as SI	Predicted as LA	Correct Prediction %	Fisher’s Prob.
M1	SI	34	32	2	97.62	3.6 × 10^−21^
	LA	50	0	50		
M2	SI	19	16	3	90.48	8.8 × 10^−8^
	LA	23	1	22		
M3	SI	19	16	3	92.86	5.8 × 10^−9^
	LA	23	0	23		
M4	SI	26	20	6	87.3	3 × 10^−9^
	LA	37	2	35		
M5	SI	26	15	11	80.95	7.9 × 10^−7^
	LA	37	1	36		
M6	SI	26	15	11	80.95	7.9 × 10^−7^
	LA	37	1	36		
M7	SI	78	60	18	85.19	1.5 × 10^−22^
	LA	111	10	101		
M8	SI	26	20	6	85.71	2.0 × 10^−8^
	LA	37	3	34		
M9	SI	26	20	6	85.71	2.0 × 10^−8^
	LA	37	3	34		

## Data Availability

The original contributions presented in the study are included in the article/Supplementary Material, further inquiries can be directed to the corresponding author.

## Supplementary Materials

The following supporting information can be downloaded at: https://www.mdpi.com/article/10.3390/molecules29184441/s1, Figure S1: 1D ^1^H NOESY spectra of aqueous extracts of squeezed tomato (P1), lyophilized tomato (P2), and homogenized tomato (P3); Table S1: List of metabolites contained in the aqueous extracts of tomato and identified via 1D ^1^H NOESY measurements; Figure S2: A portion of the ^1^H-^15^N HMQC of a reference solution of glutamine at pH 4.2; Figure S3: ^1^H-NOESY/EXSY spectrum of a reference solution of glutamine at pH 4.2; Figure S4: ^1^H-NOESY/EXSY spectrum of aqueous extracts of tomato; Figure S5: Hierarchical clustering dendrogram; Figure S6: Correlation heatmap; Table S2: Correlation table; Figure S7: Results of the permutation tests; Figure S8: Prediction scores plot (P1/O1) of OPLS-DA models (a) M1, (b) M2, (c) M3, (d) M4, (e) M5, (f) M6, (g) M7, (h) M8, and (i) M9.
