# Supplementary material for: Non-Targeted Nuclear Magnetic Resonance Analysis for Food Authenticity: A Comparative Study on Tomato Samples
Source: Molecules. 2024 Sep 19;29(18):4441. doi: 10.3390/molecules29184441 (PMC11434360; doi:10.3390/molecules29184441)

Supporting Information

Non-Targeted Nuclear Magnetic Resonance Analysis for Food Authenticity: A Comparative Study on Tomato Samples

Biagia Musio [1,*], Rosa Ragone [1], Stefano Todisco [1], Antonino Rizzuti [1], Egidio Iorio [2], Mattea Chirico [2], Maria Elena Pisanu [2], Nadia Meloni [3], Piero Mastrorilli [1,4] and Vito Gallo [1,4]

[1] Department of Civil, Environmental, Land, Building Engineering and Chemistry (DICATECh), Polytechnic University of Bari, Via Orabona, 4, I-70125 Bari, Italy; rosa.ragone@poliba.it (R.R.); stefano.todisco@poliba.it (S.T.); antonino.rizzuti@poliba.it (A.R.); piero.mastrorilli@poliba.it (P.M.); vito.gallo@poliba.it (V.G.)

[2] Istituto Superiore di Sanità, Core Facilities Istituto Superiore Di Sanità, Viale Regina Elena, 299, I-00161 Roma, Italy; egidio.iorio@iss.it (E.I.); mattea.chirico@iss.it (M.C.); mariaelena.pisanu@iss.it (M.E.P.)

[3] Agenzia Regionale Protezione Ambientale Lazio, Dipartimento Prevenzione e Laboratorio Integrato, Servizio Coordinamento delle Attività di Laboratorio, Unità Laboratorio Chimico di Latina, Via Mario Siciliano, 1, I-04100 Latina , Italy; nadia.meloni@arpalazio.it

[4] Innovative Solutions S.r.l.,Spin-Off Company of the Polytechnic University of Bari, Zona H 150/B, I-70015 Noci, Italy

* Correspondence: biagia.musio@poliba.it; Tel.: +39-0805963569

Sommario

Figure S1. 1D ^{1}H NOESY spectra of aqueous extracts of squeezed tomato (P1), lyophilized tomato (P2), and homogenized tomato (P3). 3

Table S1. List of metabolites contained in the aqueous extracts of tomato and identified via a typical 1D ^{1}H NOESY spectrum. 4

Figure S2. A portion of the ^{1}H-^{15}N HMQC of a reference solution of glutamine at pH 4.2. 5

Figure S3. ^{1}H-NOESY/EXSY spectrum of a reference solution of glutamine at pH 4.2. 6

Figure S4. ^{1}H-NOESY/EXSY spectrum of aqueous extracts of tomato. 7

Figure S5. Hierarchical clustering dendrogram. 8

Figure S6. Correlation heatmap. 9

Table S2. Correlation table. 10

Figure S7. Results of the permutation tests. 11

Figure S8. Prediction scores plot (P1/O1) of OPLS-DA models a) M1, b) M2, c) M3, d) M4, e) M5, f) M6, g) M7, h) M8, and i) M9. 14

Figure S1. 1D ^{1}H NOESY spectra of aqueous extracts of squeezed tomato (P1), lyophilized tomato (P2), and homogenized tomato (P3).

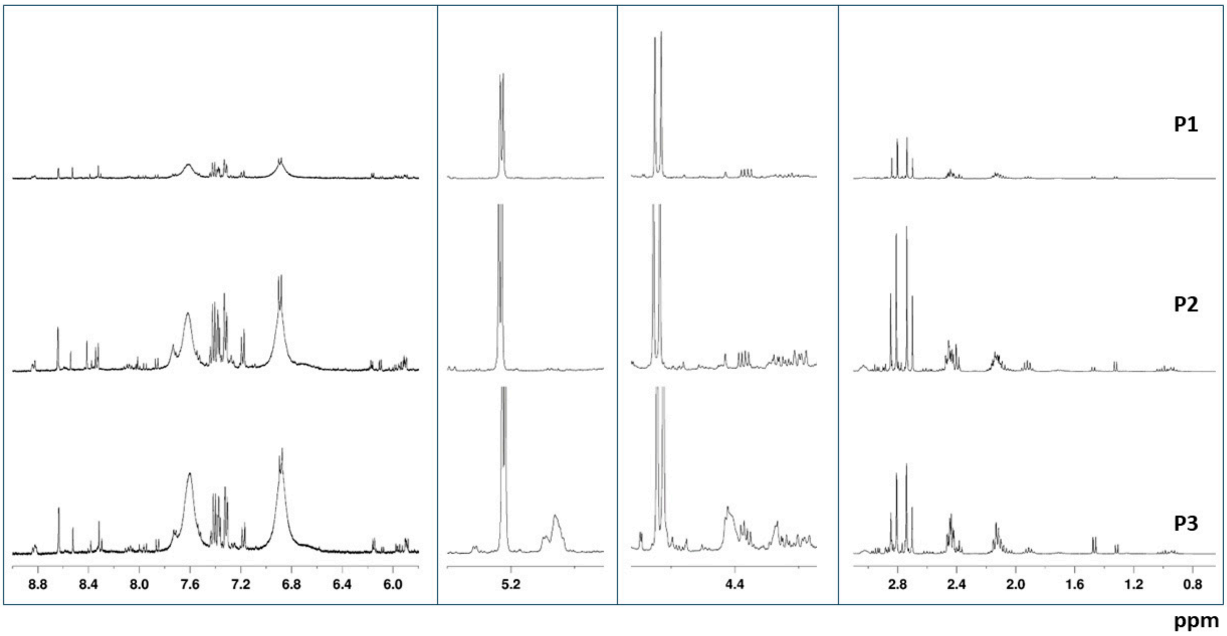

Table S1. List of metabolites contained in the aqueous extracts of tomato and identified via a typical 1D ^{1}H NOESY spectrum.

Compound n.	Compound	Chemical shift (ppm)	Assignment	Signal multiplicity (coupling constant, Hz)
1	Isoleucine	0.93	$C_{\delta}H_3$	t (7.4)
		1.00	$C_{\gamma}H_3$	d (7.3)
2	Leucine	0.95	$C_{\delta'}H_3 + C_{\delta}H_3$	m[a]
		1.71	$C_{\beta}H_2 + C_{\gamma}H$	m
3	Valine	0.98	$C_{\gamma}H_3$	d (7.0)
		1.03	$C_{\gamma'}H_3$	d (7.0)
4	Ethanol	1.17	CH_3	t (7.0)
5	Threonine	1.32	$C_{\gamma}H_3$	d (6.7)
6	Alanine	1.47	$C_{\beta}H_3$	d (7.2)
7	GABA	1.92	$C_{\beta}H_2$	quintet (7.5)
		2.39	$C_{\alpha}H_2$	t (7.5)
		3.03	$C_{\gamma}H_2$	br s
8	Acetate	2.04	CH_3	s
9	Glutamine	2.12	$C_{\beta}H$	m
		2.45	$C_{\gamma}H$	m
		6.88	$HN_{\varepsilon}H$	br s
		7.60	$HN_{\varepsilon}H$	br s
10	Malic Acid	2.60	H-C-*H*	dd (15.6; 8.3)
		2.80	*H*-C-H	dd (7.5; 4.3)
		4.37	HO-C-*H*	dd (8.3; 4.3)
11	Citric Acid	2.73	HC-*H*	d (15.7)
		2.83	*H*-CH	d (15.7)
12	Aspartic Acid	2.85	H-C_{β}–*H*	dd (17.5; 7.5)
		2.95	*H*-C_{β}–H	dd (17.5; 4.0)
13	Choline	3.19	N-CH_3	s
14	Glucose	5.23	α-Glu C(1)H	d (3.7)
		4.64	β-Glu C(1)H	d (7.9)
		3.24	β-Glu C(2)H	dd (8.6; 8.1)
15	Methanol	3.35	CH_3	s
16	Fructose	From 3.98 to 4.04	β–pyr C(5)H + β–pyr C(6)H	m
		4.10	β-fur C(3)H + β-fur C(4)H	m
17	Poly-galacturonate	5.07		m
		4.43		m
18	Galacturonate	5.29	α-Pyr C(1)H	d (3.9)
		4.57	β-Pyr C(1)H	d (8.1)
19	Uridine	5.89	C(5)-H	d (8.3)
		5.89	Rib C(1)-H	d (4.7)
		7.86	C(6)-H	d (8.3)
20	Adenosine	6.09	Rib C(1)-H	d (5.9)
		8.27	C2-*H*	s
		8.40	C8-*H*	s
21	Tyrosine	6.88	*ortho*-Ph(H)	d (8.3)
		7.18	*meta*-Ph(H)	d (8.3)
22	Phenylalanine	7.40	*ortho*-Ph(H)	m
		7.32	*meta*+*para*-Ph(H)	m
23	Trigonelline	8.83	C(3)-H + (C5)-H	br s
		9.12	C(1)-H	s

[a] A_3B_3 system that appears as a pseudo doublet of doublets.

Figure S2. A portion of the ^{1}H-^{15}N HMQC of a reference solution of glutamine at pH 4.2.

A tube was prepared dissolving 15 mg of pure glutamine in 540 µL di oxalate buffer and 60 µL of D_2O and 0.2 % of TSP-d_4. Red peaks indicate the correlation peaks due to scalar coupling, using a constant J-Coupling of 90Hz, without decoupling.

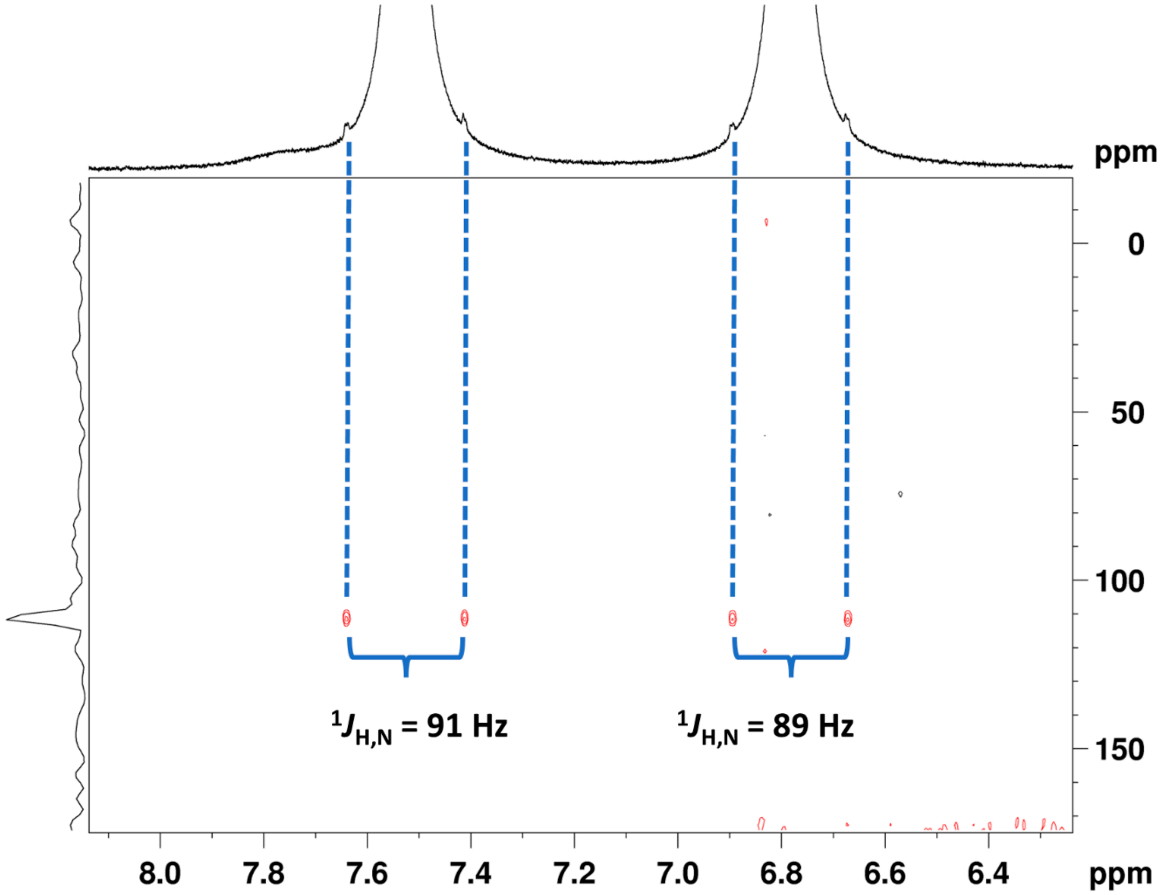

Figure S3. ^{1}H-NOESY/EXSY spectrum of a reference solution of glutamine at pH 4.2.

A tube was prepared dissolving 15 mg of pure glutamine in 540 µL di oxalate buffer and 60 µL of D_2O and 0.2 % of TSP-d_4. The experiment was conducted at 298.2 K with a mixing time of 1.0 s. Blue peaks are positive indicating peaks of exchange, and red peaks are negative indicating NOE contacts between protons close in space.

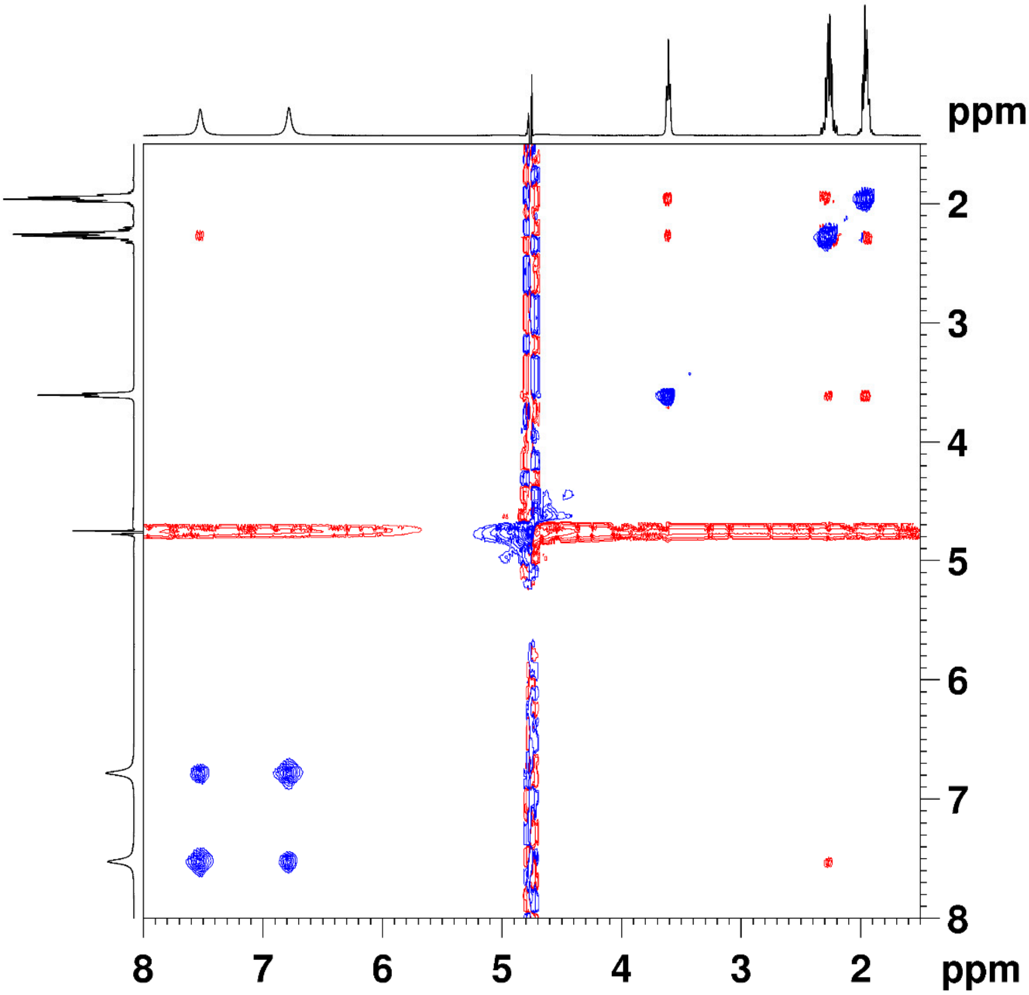

Figure S4. ^{1}H-NOESY/EXSY spectrum of aqueous extracts of tomato.

Measurement was acquired at 298.2 K with a mixing time of 1.0 s. Blue peaks are positive indicating peaks of exchange, and red peaks are negative indicating NOE contacts between protons close in space.

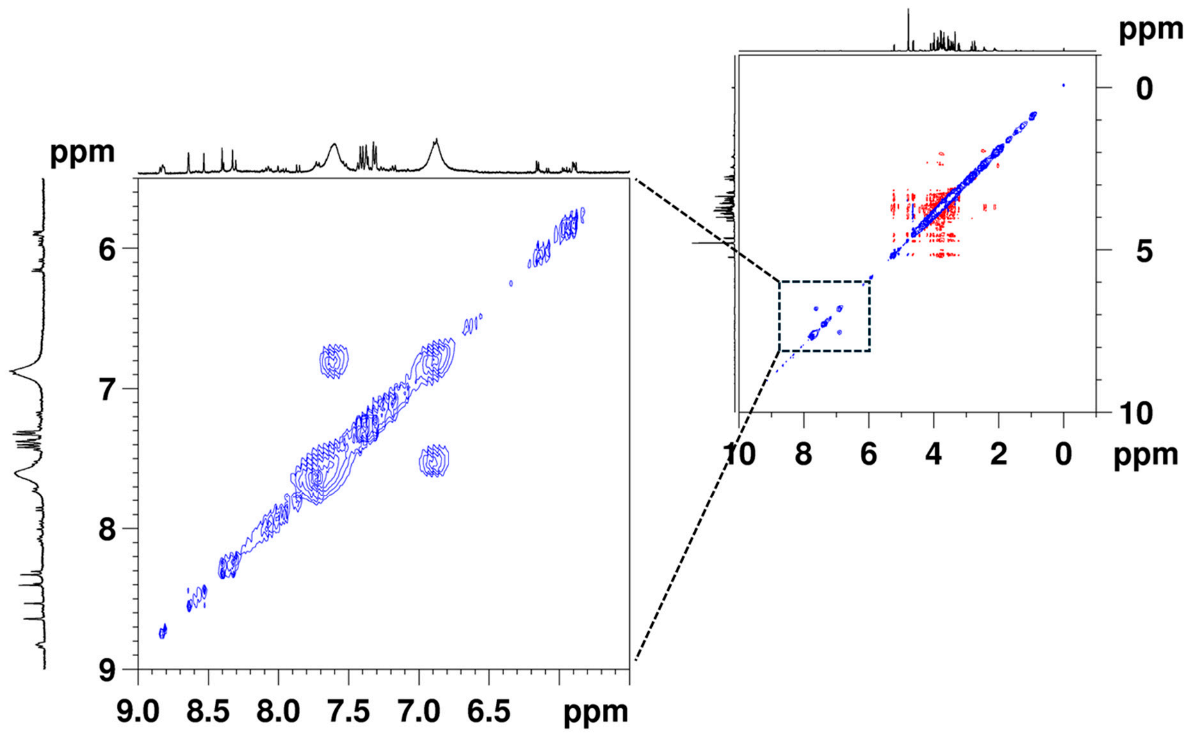

Figure S5. Hierarchical clustering dendrogram.

The clustering result is shown as a dendrogram (distance measure using Euclidean as distance measure and ward as a clustering algorithm (clustering uses the centroids of the observations).

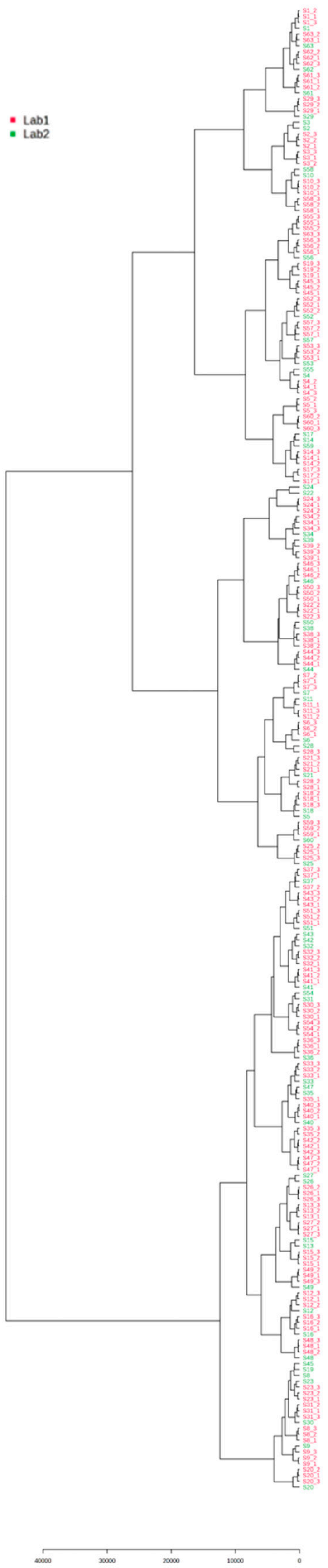

Figure S6. Correlation heatmap.

The correlation results are shown as a heatmap. The correlations are colored according to the values of the correlation coefficient as indicated in the lateral bar.

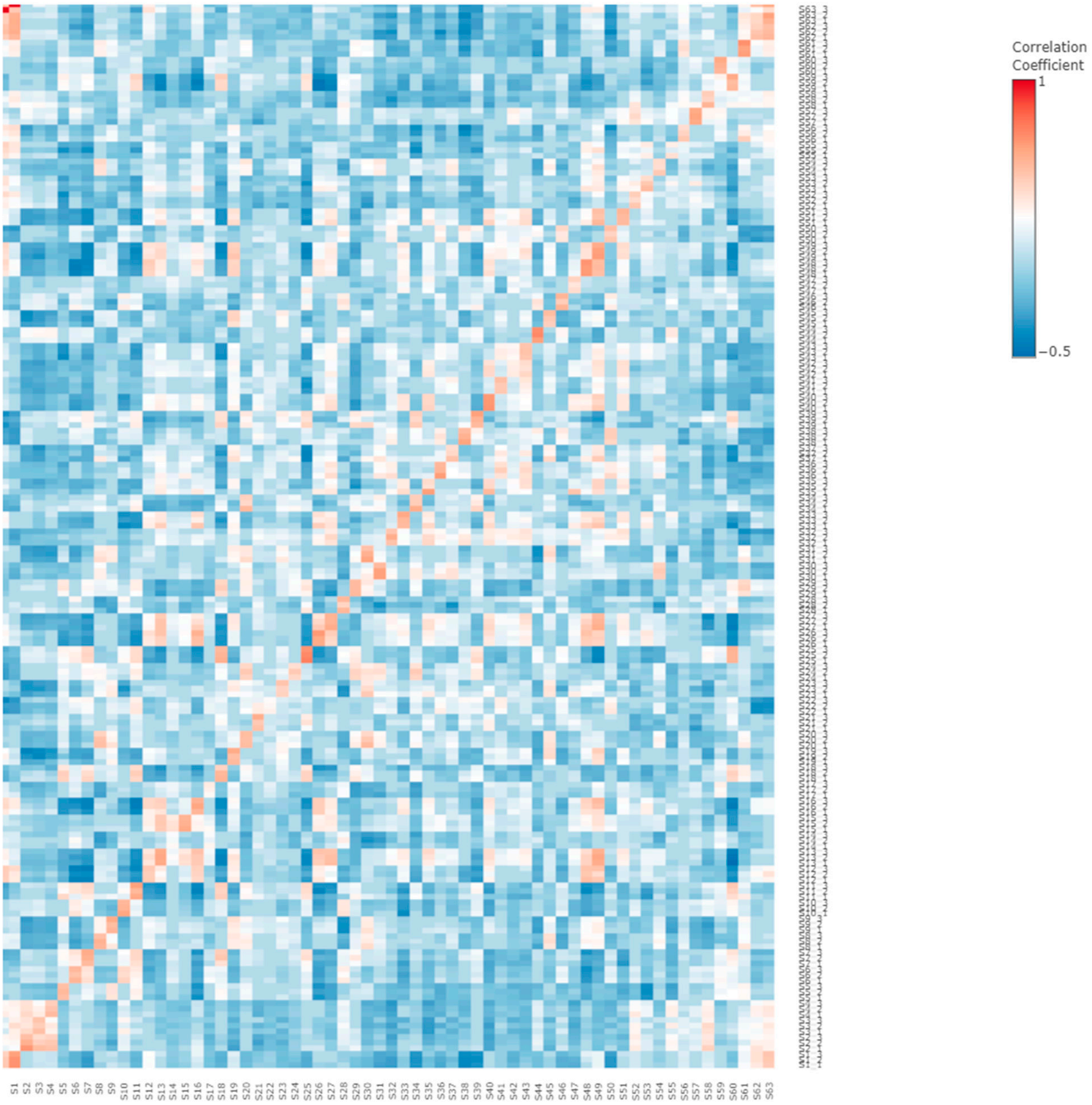

Table S2. Correlation table.

The Kendall rank correlations are indicated according to the coefficient value, i.e. values >0.50 in red and 0.45<values<0.50 in orange.

	S1	S2	S3	S4	S5	S6	S7	S8	S9	S10	S11	S12	S13	S14	S15–S63
S1_1	0,67	0,00	0,06	0,06	-0,12	-0,21	-0,33	-0,08	0,07	0,00	-0,20	0,22	0,00	-0,13	[illegible]
S1_2	0,67	0,00	0,07	0,06	-0,16	-0,23	-0,34	0,00	0,00	-0,07	-0,22	0,22	0,00	-0,11	[illegible]
S1_3	0,62	0,00	0,08	0,05	-0,11	-0,22	-0,30	0,00	0,06	-0,07	-0,20	0,23	0,07	-0,11	[illegible]
S2_1	0,22	0,65	0,51	0,48	-0,10	0,14	0,00	-0,08	-0,20	0,17	0,00	0,00	0,00	-0,10	[illegible]
S2_2	0,26	0,56	0,43	0,46	-0,20	0,11	0,00	-0,13	-0,29	0,15	-0,08	0,00	0,00	-0,15	[illegible]
S2_3	0,29	0,50	0,37	0,41	-0,17	0,11	0,00	-0,06	-0,20	0,15	0,00	0,00	-0,08	-0,13	[illegible]
S3_1	0,29	0,38	0,49	0,28	-0,08	0,12	0,00	0,00	-0,16	0,29	0,11	0,00	-0,16	-0,09	[illegible]
S3_2	0,37	0,33	0,44	0,32	-0,12	0,09	-0,06	-0,08	-0,19	0,14	0,00	0,00	-0,13	-0,16	[illegible]
S3_3	0,28	0,34	0,48	0,24	-0,09	0,07	0,00	-0,07	-0,16	0,26	0,09	-0,05	-0,16	-0,11	[illegible]
S4_1	0,26	0,39	0,31	0,51	-0,09	0,07	0,00	-0,09	-0,18	0,12	0,00	0,07	-0,09	-0,12	[illegible]
S4_2	0,27	0,35	0,29	0,47	-0,11	0,00	0,00	-0,11	-0,20	0,09	-0,06	0,00	-0,10	-0,16	[illegible]
S4_3	0,29	0,35	0,24	0,43	-0,16	0,07	0,00	-0,18	-0,24	0,18	-0,13	0,00	-0,09	-0,16	[illegible]
S5_1	0,00	-0,11	-0,15	-0,17	0,49	0,08	0,20	0,00	0,33	0,00	0,27	-0,15	-0,22	0,00	[illegible]
S5_2	0,00	-0,11	-0,12	-0,15	0,52	0,00	0,19	0,07	0,34	0,00	0,29	-0,09	-0,17	0,00	[illegible]
S5_3	0,00	-0,16	-0,18	-0,22	0,49	0,00	0,15	0,06	0,34	0,00	0,25	-0,10	-0,18	0,00	[illegible]
S6_1	-0,10	0,09	0,09	0,00	0,09	0,54	0,36	-0,15	-0,14	0,41	0,25	-0,24	-0,15	0,15	[illegible]
S6_2	0,00	0,06	0,06	-0,07	0,08	0,48	0,33	-0,14	-0,11	0,37	0,28	-0,24	-0,18	0,11	[illegible]
S6_3	-0,08	0,13	0,11	0,00	0,08	0,53	0,34	-0,18	-0,17	0,39	0,32	-0,29	-0,19	0,09	[illegible]
S7_1	-0,17	0,00	0,00	-0,07	0,19	0,28	0,52	0,00	0,00	0,19	0,36	-0,23	-0,20	0,07	[illegible]
S7_2	-0,21	0,06	0,00	-0,05	0,25	0,31	0,56	0,00	0,00	0,19	0,39	-0,26	-0,19	0,14	[illegible]
S7_3	-0,23	0,07	0,00	0,00	0,24	0,34	0,58	-0,08	-0,06	0,23	0,40	-0,29	-0,26	0,11	[illegible]
S8_1	0,15	-0,26	-0,24	-0,27	0,12	-0,28	-0,17	0,53	0,32	-0,16	0,00	0,20	0,07	0,00	[illegible]
S8_2	0,12	-0,25	-0,23	-0,21	0,00	-0,31	-0,20	0,55	0,22	-0,16	0,00	0,20	0,05	0,00	[illegible]
S8_3	0,17	-0,25	-0,23	-0,23	0,00	-0,27	-0,17	0,49	0,21	-0,18	0,00	0,16	0,00	-0,08	[illegible]
S9_1	0,06	-0,31	-0,26	-0,28	0,19	-0,24	-0,11	0,27	0,53	-0,18	0,00	0,19	0,17	0,00	[illegible]
S9_2	0,08	-0,30	-0,27	-0,28	0,19	-0,28	-0,13	0,27	0,51	-0,24	0,00	0,22	0,16	0,00	[illegible]
S9_3	0,11	-0,33	-0,26	-0,27	0,27	-0,25	-0,12	0,27	0,58	-0,27	0,00	0,17	0,10	0,00	[illegible]
S10_1	0,00	0,12	0,15	0,00	0,00	0,34	0,21	-0,12	-0,17	0,60	0,18	-0,19	-0,19	0,00	[illegible]
S10_2	0,06	0,07	0,11	0,00	0,00	0,28	0,18	-0,14	-0,22	0,55	0,15	-0,18	-0,18	0,00	[illegible]
S10_3–S63_3	[illegible]	[illegible]	[illegible]	[illegible]	[illegible]	[illegible]	[illegible]	[illegible]	[illegible]	[illegible]	[illegible]	[illegible]	[illegible]	[illegible]	[illegible]

Figure S7. Results of the permutation tests.

OPLS-DA model validation by permutation tests based on prediction accuracy OPLS-DA models a-b) M1, c-d) M2, e-f) M3, g-h) M4, i-j) M5, k-l) M6, and m-n) M7, for Sicily (SI) and Lazio (LA) samples, respectively.

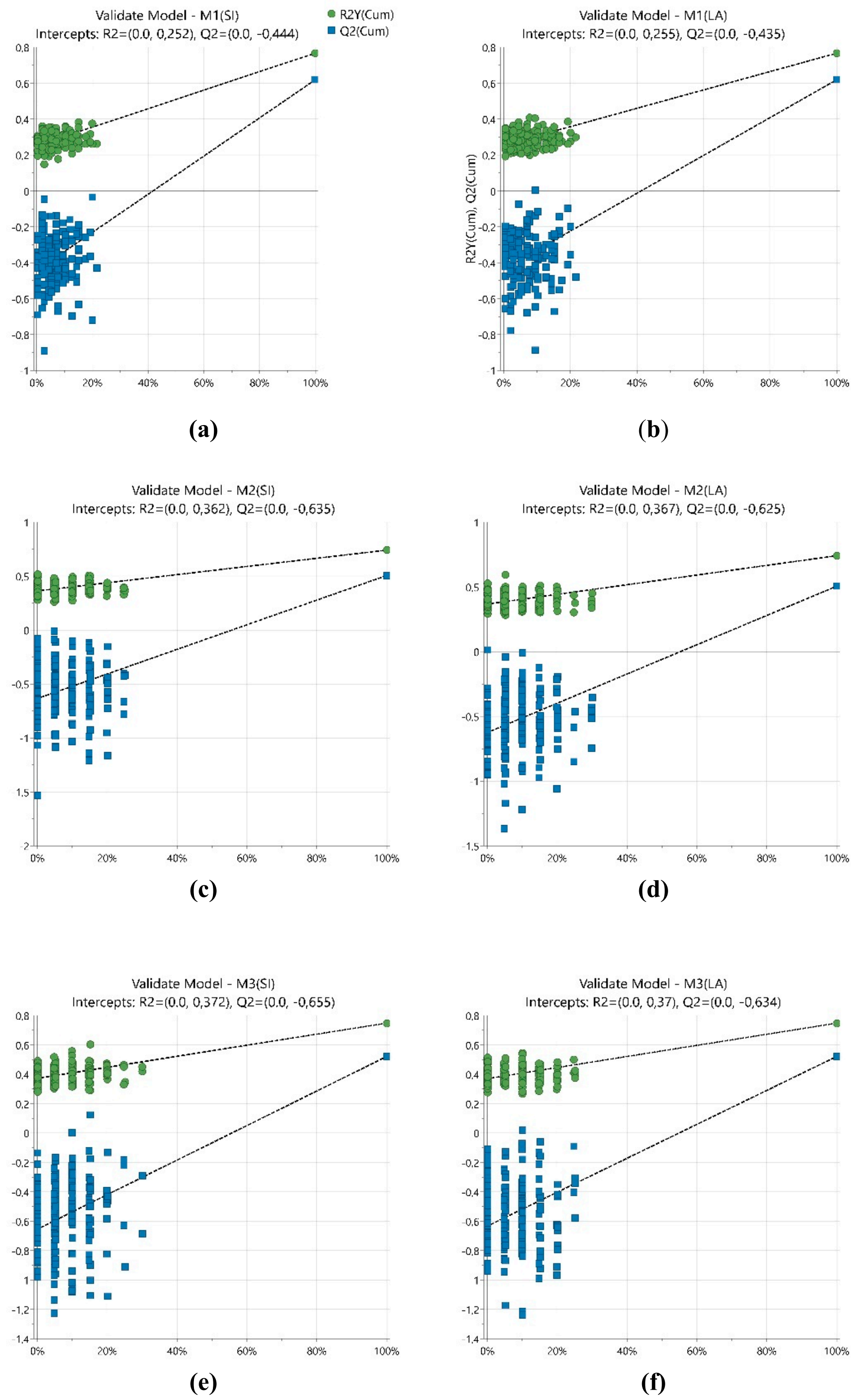

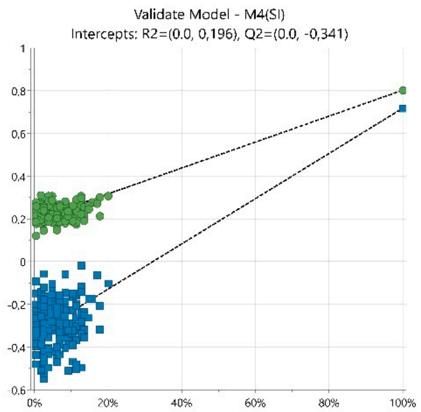
Validate Model - M4(SI)
Intercepts: R2=(0.0, 0,196), Q2=(0.0, -0,341)
1
0,8
0,6
0,4
0,2
0
-0,2
-0,4
-0,6
0%
20%
40%
60%
80%
100%

(g)

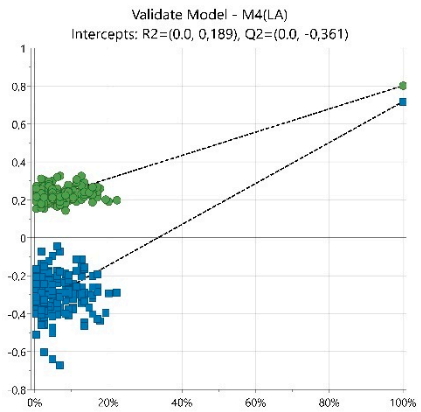
Validate Model - M4(LA)
Intercepts: R2=(0.0, 0,189), Q2=(0.0, -0,361)
1
0,8
0,6
0,4
0,2
0
-0,2
-0,4
-0,6
-0,8
0%
20%
40%
60%
80%
100%

(h)

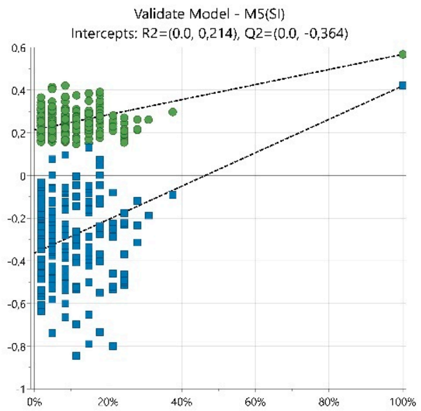
Validate Model - M5(SI)
Intercepts: R2=(0.0, 0,214), Q2=(0.0, -0,364)
0,6
0,4
0,2
0
-0,2
-0,4
-0,6
-0,8
-1
0%
20%
40%
60%
80%
100%

(i)

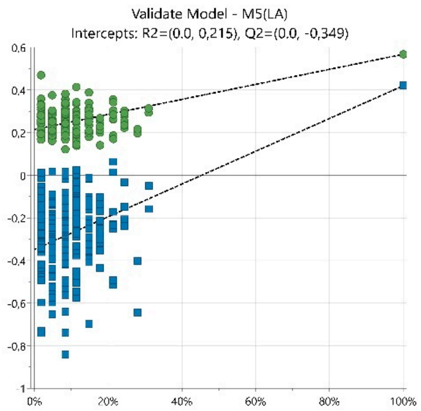
Validate Model - M5(LA)
Intercepts: R2=(0.0, 0,215), Q2=(0.0, -0,349)
0,6
0,4
0,2
0
-0,2
-0,4
-0,6
-0,8
-1
0%
20%
40%
60%
80%
100%

(j)

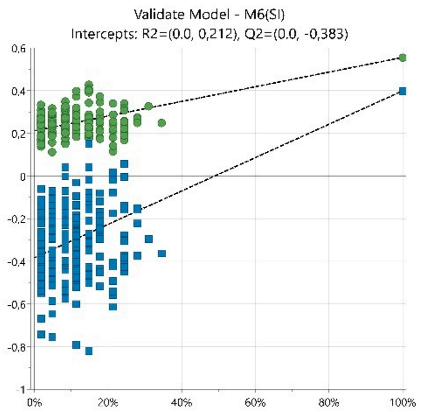
Validate Model - M6(SI)
Intercepts: R2=(0.0, 0,212), Q2=(0.0, -0,383)
0,6
0,4
0,2
0
-0,2
-0,4
-0,6
-0,8
-1
0%
20%
40%
60%
80%
100%

(k)

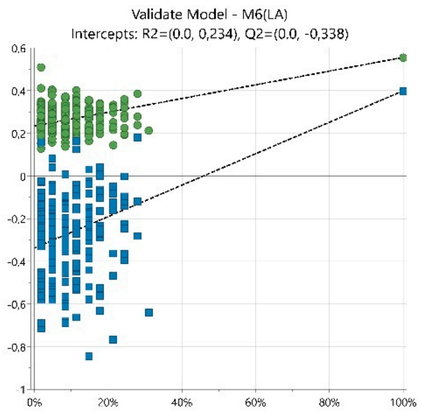
Validate Model - M6(LA)
Intercepts: R2=(0.0, 0,234), Q2=(0.0, -0,338)
0,6
0,4
0,2
0
-0,2
-0,4
-0,6
-0,8
-1
0%
20%
40%
60%
80%
100%

(l)

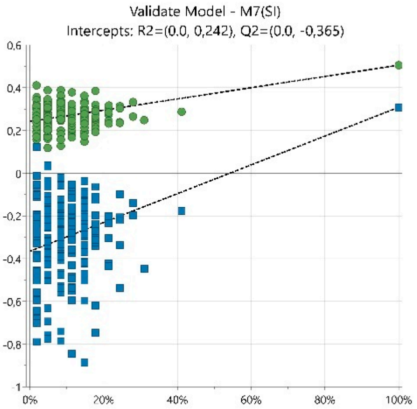
Validate Model - M7(SI)
Intercepts: R2=(0.0, 0,242), Q2=(0.0, -0,365)
0,6
0,4
0,2
0
-0,2
-0,4
-0,6
-0,8
-1
0%
20%
40%
60%
80%
100%

(m)

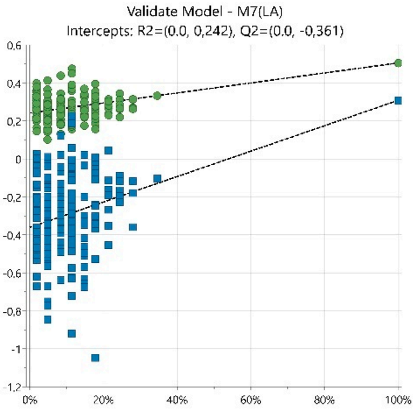
Validate Model - M7(LA)
Intercepts: R2=(0.0, 0,242), Q2=(0.0, -0,361)
0,6
0,4
0,2
0
-0,2
-0,4
-0,6
-0,8
-1
-1,2
0%
20%
40%
60%
80%
100%

(n)

Figure S8. Prediction scores plot (P1/O1) of OPLS-DA models a) M1, b) M2, c) M3, d) M4, e) M5, f) M6, g) M7, h) M8, and i) M9.

The observations are indicated as circles and inverted triangles when they are included in the work set and the prediction set, respectively. The observations are indicated as green scores and blue scores, according to the geographical origin, i.e. Sicily and Lazio, respectively. The ellipse shows the 95% confidence interval using Hotelling T2 statistics.

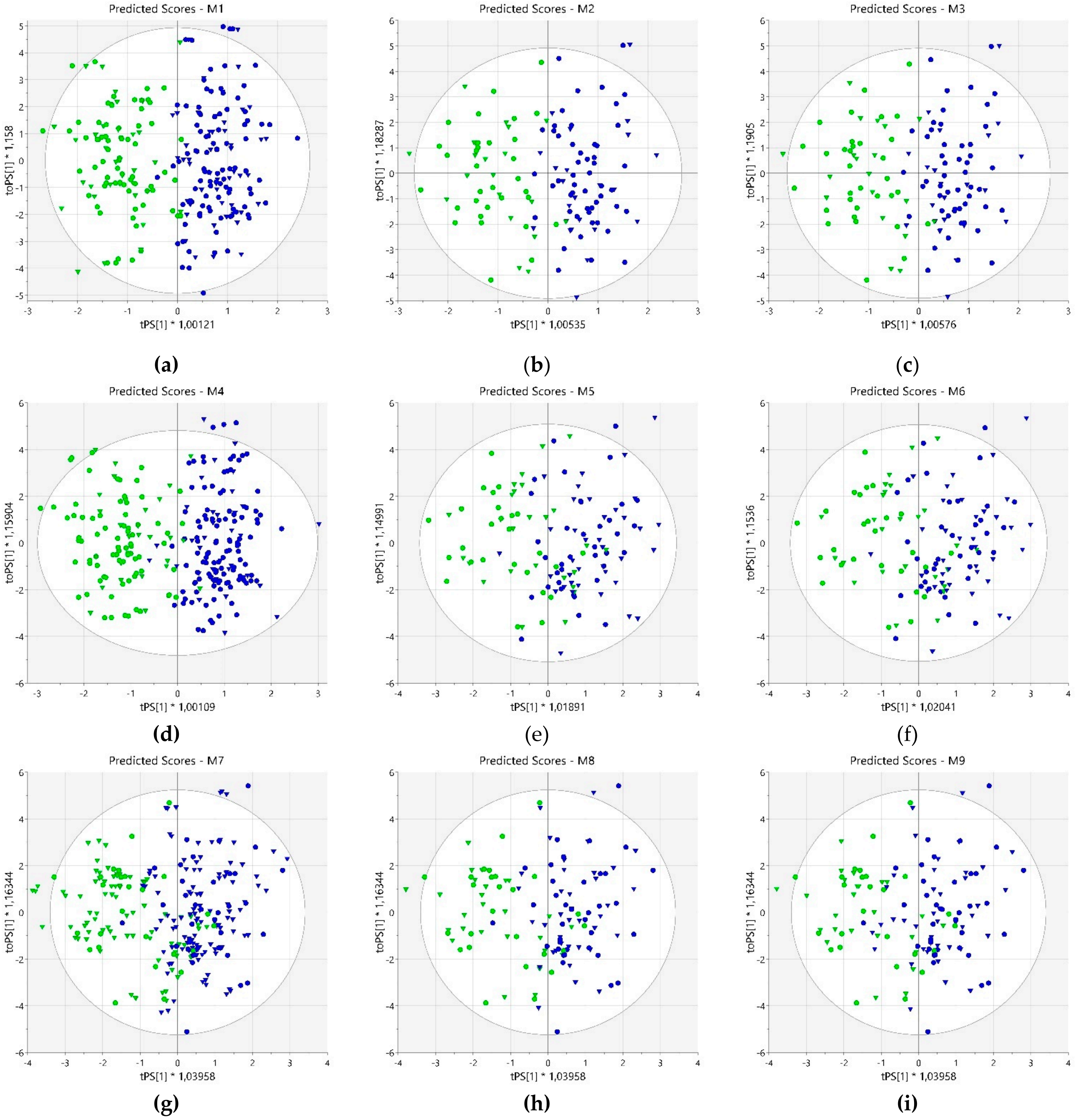

Supplement: Supplementary file 1 [file molecules-29-04441-s001.zip › molecules-3174172-supplementary.pdf]
